# Human reconstruction using 3D Gaussian Splatting: a brief survey

**DOI:** 10.3389/frai.2025.1709229

**Published:** 2025-11-21

**Authors:** Dong-Lin Chen, Mohd Shafry Mohd Rahim, Hiew Moi Sim, Bin Wang, Si Chen, Min-Song Li

**Affiliations:** 1 Faculty of Computing, Universiti Teknologi Malaysia, Johor Bahru, Malaysia; 2 School of Big Data Science, Jiangxi Institute of Fashion Technology, Nanchang, China; 3 Faculty of Computing and Information Technology, Sohar University, Sohar, Oman; 4 School of Information Engineering, ShaoGuan University, ShaoGuan, China

**Keywords:** 3D Gaussian Splatting, human reconstruction, human template, SMPL, animatable avatar

## Abstract

Reconstructing high-fidelity and animatable 3D human avatars from visual data is a core task for immersive applications such as virtual reality (VR) and digital content creation. While traditional approaches often suffer from high computational costs, slow inference, and visual artifacts, recent advances leverage 3D Gaussian Splatting (3DGS) to enable rapid training and real-time rendering (up to 361 FPS). A common framework leverages parametric models to establish a canonical human representation, followed by deformation of 3D Gaussians into target poses using learnable skinning and novel regularization techniques. Key advances include deformation mechanisms for motion generalization, hybrid Gaussian-mesh representations for complex clothing and geometry, efficient compression and acceleration strategies, and specialized modules for handling occlusions and fine details. This article briefly reviews recent progress in 3DGS-based human reconstruction, we organize methods by input type: single-view and multi-view reconstruction. We discuss the strengths and limitations of each category and highlight promising future directions.

## 1 Introduction

The creation of high-fidelity animatable 3D human avatars is a fundamental objective in computer vision and graphics, with broad applications in VR and digital content creation. Despite significant advances, faithfully reconstructing dynamic humans with varied clothing from single-view or multi-view data remains challenging. Articulated motion, non-rigid deformations, occlusions, and the need for real-time performance impose stringent demands on reconstruction systems.

Traditional 3D reconstruction methods typically rely on specialized hardware such as 3D scanning chambers. With the advent of deep learning, data-driven approaches have emerged that reconstruct 3D human shapes directly from RGB inputs (e.g., single images, multi-view images). For instance, PIFu (Saito et al., 2019) predicts 3D occupancy fields from aligned image features and extracts meshes via marching cubes (Lorensen and Cline, 1987). To improve reconstruction robustness, many recent methods incorporate parametric human models like SMPL (Loper et al., 2015) and SMPL-X (Pavlakos et al., 2019). Representative works include ARCH (Huang et al., 2020), ARCH++ (He et al., 2021), ICON (Xiu et al., 2022), CAR (Liao et al., 2023), VINECS (Liao et al., 2024), and CanonicalFusion (Shin et al., 2025). More recently, methods such as SiTH (Ho et al., 2024), PSHuman (Li et al., 2025), and PARTE (Nam et al., 2025) integrate diffusion models to infer occluded views, thereby enhancing both geometric detail and visual appearance. Despite these advances, a noticeable gap remains between current reconstruction accuracy and the demands of real-world applications. Concurrently, neural implicit representations like Neural Radiance Fields (NeRFs) (Mildenhall et al., 2022) improve the visual quality in novel view synthesis. However, their high computational burden and slow rendering speeds often limit their practicality for reconstructing and animating human subjects. In contrast, 3D Gaussian Splatting (3DGS) (Kerbl et al., 2023) introduces an explicit and differentiable representation that achieves state-of-the-art visual quality while enabling fast training (often under 1.5 h) and real-time rendering, marking a significant shift from previous paradigms.

A dominant framework in 3DGS-based human reconstruction deforms canonical 3D Gaussians into target poses using learned skinning mechanisms, heavily leveraging SMPL-based priors. Recent efforts have extended this core idea across several dimensions: (i) novel deformation techniques using MLPs, graph networks, or attention mechanisms improve motion generalization; (ii) hybrid representations combine Gaussians with explicit surfaces (meshes, tetrahedra, or surfels) for complex cloth and topological detail; (iii) efficient compression and rasterization strategies enable deployment on consumer hardware; and (iv) specialized modules address persistent challenges such as occlusion handling, facial animation, and fine-grained dynamic details. This article surveys recent progress in 3DGS for human reconstruction, organizing methods by input modality: single-view and multi-view setups. We discuss representative works, analyze their trade-offs between speed, fidelity, and generality, and identify promising future research directions.

## 2 3D Gaussian Splatting

3DGS (Kerbl et al., 2023) represents 3D data using a set of discrete geometric primitives known as 3D Gaussians. Each Gaussian is defined by a center position μ ∈ ℝ^3^, a scaling vector **s** ∈ ℝ^3^, and a rotation quaternion **q** ∈ ℝ^4^. These parameters are used to construct a covariance matrix **Σ** ∈ ℝ^3 × 3^ in a physically plausible manner as: **Σ** = *RSS*^*T*^*R*^*T*^, where **S** is the scaling matrix, and **R** is the rotation matrix derived from **q**. To model appearance, each Gaussian is associated with an opacity value α ∈ [0, 1] and view-dependent color properties **c** ∈ ℝ^*C*^ represented via spherical harmonics coefficients. During rendering, the 3D Gaussians are projected onto the 2D image plane as splats. A tile-based rasterizer is employed to efficiently combine contributions from all splats overlapping a pixel.

**Figure 1:**
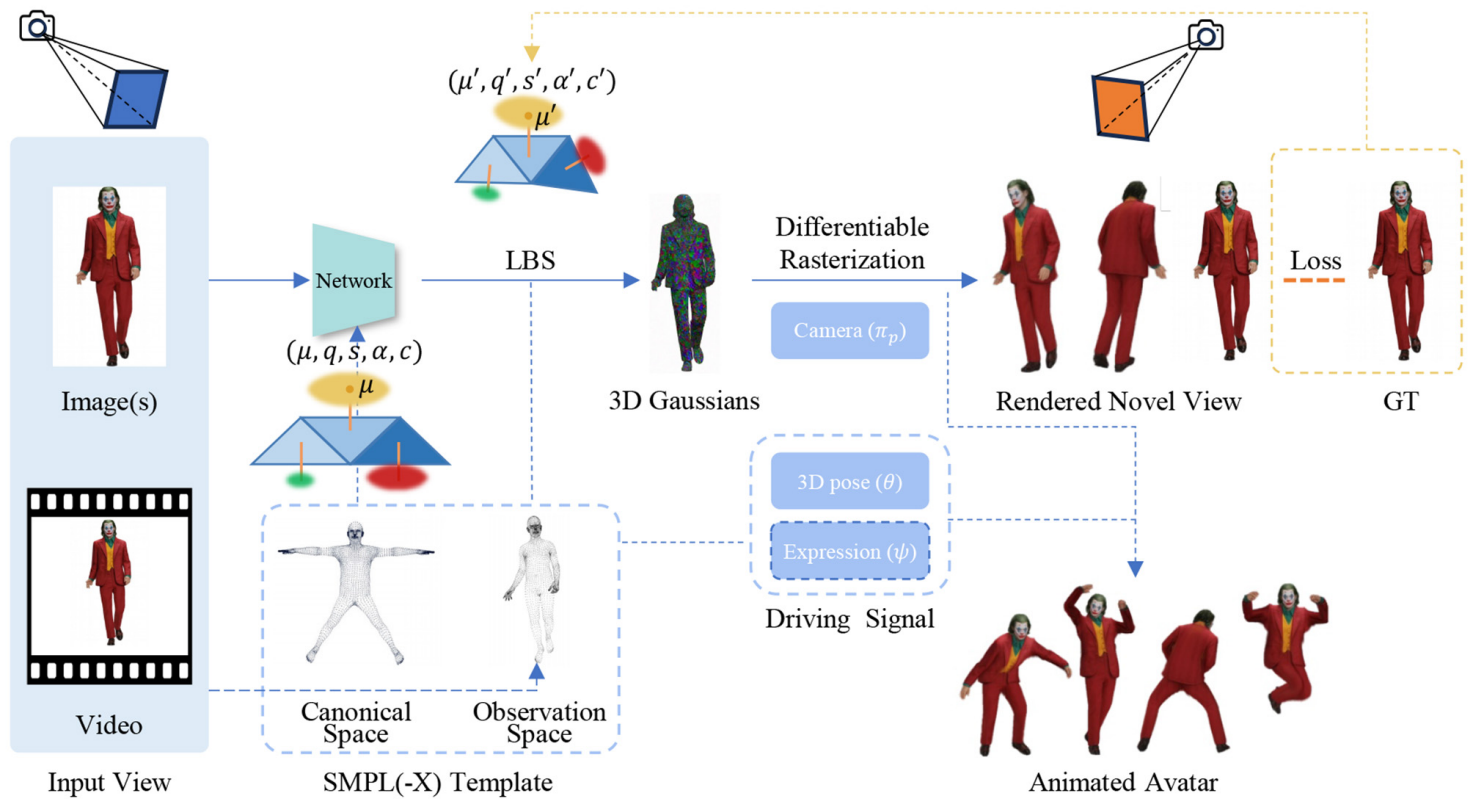
Overview of the 3DGS human reconstruction pipeline. The core objective of 3DGS avatar generation methods is to train a network to accurately predict the parameters of 3D Gaussians, denoted as *G*(μ, *q, s*, α, *c*). The pipeline typically starts by initializing a point cloud from the vertices of an SMPL(-X) model. The positions and rotations of the Gaussians are then transformed into the observation space via forward linear blend skinning (LBS). Differentiable rasterization is subsequently applied to render the target novel view image. The resulting animatable avatars can be driven by pose sequences and expression signals (if applicable). Part of image source generated by LHM (Qiu et al., 2025a).

3DGS provides an explicit and fully differentiable representation that is particularly suitable for modeling dynamic human subjects. A highly influential paradigm adopted by many recent methods involves establishing 3D Gaussians in a canonical space and deforming them into target poses using learned skinning fields, leveraging strong priors from parametric human templates (e.g., SMPL, SMPL-X), as depicted in Figure 1. In terms of implementation, optimization-based methods for 3DGS human reconstruction iteratively optimizes Gaussian parameters to minimize a rendering loss, a process that is relatively intensive in computation. On the other hand, LHM (Qiu et al., 2025a) exemplifies the feed-forward paradigm, using a network to generate animatable 3D avatars from a single image in seconds, thereby bypassing the costly optimization loop.

## 3 3DGS-based human reconstruction

The emergence of 3DGS has introduced a significant shift in the field of 3D human reconstruction, effectively bridging the long-standing gap between high-fidelity rendering and real-time performance. This section systematically reviews these advancing techniques, organizing them according to input type: single-view and multi-view reconstruction, while critically examining core innovations in deformation modeling, hybrid representation design, and regularization strategies that facilitate robust animation and generalization. The 3DGS-based human reconstruction methods are summarized in Table 1.

**Table 1:** Summary of 3DGS-based human reconstruction methods.

**Method**	**Publication**	**Input**	**GPU**	**Training**	**FPS**	**Template**	**Output**
GaussianAvatar	CVPR'24	Monocular video	1 RTX 3090	0.5~6 h	–	SMPL(-X)	Image
GauHuman	CVPR'24	Monocular video	–	1 min	189	SMPL	Image
HUGS	CVPR'24	Monocular video	1 RTX 3090Ti	8 min	60	SMPL	Image
3DGS-Avatar	CVPR'24	Monocular video	–	30 min	50	SMPL	Image
GART	CVPR'24	Monocular video	–	2.5 min	150	SMPL, SMAL	Image
SplattingAvatar	CVPR'24	Monocular video	1 RTX 3090	–	351	SMPL, FLAME	Image
GoMAvatar	CVPR'24	Monocular video	1 NVIDIA A100	–	43	SMPL	Image, Mesh
HAHA	ACCV'24	Monocular video	–	–	–	SMPL-X	Image
EVA	NeurIPS'24	Monocular video	1 NVIDIA A5000	–	361	SMPL-X	Image
StruGauAvatar	TVCG'25	Monocular video	1 RTX 3090	12 min	48	SMPL	Image, Normal
OccGaussian	ICMR'25	Monocular video	–	10	160	SMPL	Image
SGIA	TPAMI'25	Monocular video	1 RTX 3090Ti	40 min	5	SMPL	Albedo, Normal
TetGS	CVPR'25	Monocular video	1 NVIDIA A40	1.5h	–	Template-free	Mesh
HumanSplat	NeurIPS'24	Single image	8 NVIDIA A100	2 days	150	SMPL	Image
Gen-3Diffusion	TPAMI'25	Single image	8 NVIDIA A100	5 days	–	Template-free	Mesh
AniGS	CVPR'25	Single Image	–	–	–	SMPL-X	Image, Normal
Disco4D	CVPR'25	Single image	–	–	–	SMPL-X	Image
SinGS	CVPR'25	Single image	8 NVIDIA A100	–	70	SMPL	Image
HumanRef-GS	TCSVT'25	Single image	1 RTX 3090	1.5h	–	SMPL-X	Mesh
LHM	ICCV'25	Single image	64 NVIDIA A100	15.8 days	–	SMPL-X	Image
PERSONA	ICCV'25	Single image	–	–	–	SMPL-X	Image
Animatable Gaussians	CVPR'24	Multi-view video	1 RTX 4090	2 days	10	SMPL(-X)	Image
HuGS	CVPR'24	Multi-view video	1 Tesla V100	10 h	80	SMPL	Image
ASH	CVPR'24	Multi-view video	–	–	30	Habermann et al.	Image
HiFi4G	CVPR'24	Multi-view video	–	–	–	Template-free	Image
DualGS	TOG'24	Multi-view video	1 RTX 3090	–	77	Template-free	Image
LayGA	SIGGRAPH'24	Multi-view video	–	–	–	SMPL-X	Image, Normal
Anim-3D Gaussian	ACM MM'24	Multi-view video	1 RTX 3090	5 s	120	Template-free	Image
MCGS	ACM MM'24	Multi-view video	–	0.7 h	32	SMPL	Mesh, Image
SK-GS	NeurIPS'24	Multi-view video	1 Tesla V100	1.5 h	198	Template-free	Image
Hi-Fi Gaussian	CVPR'25	Multi-view video	1 RTX 3090	17.5 h	166	SMPL-X	Image
TaoAvatar	CVPR'25	Multi-view images	–	–	150	SMPL-X variant	Image
GPS-gaussian	CVPR'24	Multi-view images	–	–	25	Template-free	Image
GPS-gaussian+	TPAMI'25	Multi-view images	–	–	25	Template-free	Image
UV Gaussians	KBS'25	Multi-view images	1 NVIDIA A100	3 days	–	SMPL-X variant	Image
GBC-Splat	CVPR'25	Multi-view images	–	–	–	Template-free	Mesh
CloCap-GS	TIP'24	Multi-view images	1 RTX 2080Ti	–	–	Template-free	Mesh, Image
RoGSplat	CVPR'25	Multi-view images	1 RTX 4090	–	–	SMPL	Image

Anim-3D Gaussian means Animatable 3D Gaussian (Liu et al., 2024); “–” means no report or not applicable; Image means rendered image.

While early work on 3DGS avatar reconstruction primarily utilized monocular or multi-view video inputs, the research focus has expanded to data-efficient settings. Notably, the majority of novel works presented at top conferences and journals in 2025 predominantly employ single or sparse images as input.

### 3.1 Single-view reconstruction

#### 3.1.1 Monocular video processing

Reconstructing animatable avatars from monocular video presents a trade-off between accuracy, efficiency, and generalization. A number of methods adopt 3D Gaussian representations to achieve high-quality rendering and fast training. GaussianAvatar (Hu et al., 2024b) utilizes coarse global appearance features combined with pose information to form composite features, which are decoded into Gaussian parameters. Focusing on efficiency, GauHuman (Hu et al., 2024c) introduces canonical encoding initialized from SMPL and uses pose and linear blend skinning (LBS) refinements for deformation. It further incorporates a KL-divergence guided dynamic Gaussian control strategy (including splitting, cloning, pruning, and merging) and tile-based rasterization, achieving training in 1–2 min and rendering at 189 FPS with only 13k Gaussians. Also building on a canonical representation, HUGS (Kocabas et al., 2024) employs 3D Gaussians initialized from SMPL but allows deviations to capture loose clothing and hair. It proposes joint optimization of LBS weights to better align Gaussian motions during animation. Taking a network-based deformation approach, 3DGS-Avatar (Qian et al., 2024) combines 3DGS with a non-rigid deformable network for fast reconstruction from monocular video. It generalizes better to unseen poses through an as-isometric-as-possible regularizer applied to Gaussian means and covariances.

To enhance explicit control and structural consistency, several methods explore hybrid or template-guided Gaussian representations. GART (Lei et al., 2024) models articulated subjects using a Gaussian mixture model in canonical space, leveraging category-specific templates (e.g., SMPL/SMAL) and learnable forward skinning. It captures challenging deformations like loose clothing via a latent bone mechanism. SplattingAvatar (Shao et al., 2024) jointly optimizes Gaussian parameters and mesh embeddings directly on a mesh surface for realistic avatars. GoMAvatar (Wen et al., 2024) adopts a similar Gaussians-on-Mesh (GoM) representation, combining the rendering speed of splatting with the compatibility of mesh deformations. In a hybrid approach, HAHA (Svitov et al., 2024) attaches Gaussians to mesh polygons and uses a learned transparency map to blend splatting with mesh rendering, activating Gaussians only for complex areas like hair.

Further innovations aim to improve robustness and handling of challenging conditions such as occlusion and lighting variation. EVA (Hu et al., 2024a) proposes a context-aware density control strategy with feedback to handle varying detail levels across body parts (e.g., face vs. torso). StruGauAvatar (Zhi et al., 2025) introduces a structured Gaussian representation anchored to a DMTet (Shen et al., 2021) canonical mesh, supplemented by free Gaussians, and uses dual-space optimization to jointly refine shapes, Gaussians, and skinning weights for better generalization. For handling occlusions, OccGaussian (Ye et al., 2025) designs an occlusion-aware rendering pipeline that initializes Gaussians in canonical space and employs feature aggregation from occluded regions, enabling training from monocular occluded videos in 6 min. SGIA (Zhao et al., 2025) explores an inverse rendering approach, defining PBR-aware Gaussian attributes in canonical space and deforming them via LBS, while using an occlusion approximation to disentangle lighting and materials. These techniques highlight a diversity of strategies for overcoming the limited information in single-image inputs, though issues in pose naturalness and occlusion persist. On the other hand, TetGS (Liu et al., 2025) prioritizes editability by constraining Gaussians within a tetrahedral grid, decoupling editing into spatial adaptation and appearance learning.

#### 3.1.2 Single-image reconstruction

Reconstructing animatable humans from a single image remains challenging due to incomplete data, and is often addressed by incorporating strong generative or geometric priors. Recent advances in diffusion-based human generative models, especially those conditioned on pose, have improved model controllability and reconstruction quality. HumanSplat (Pan et al., 2024) uses a fine-tuned multi-view diffusion model to produce latent features, which are then integrated with geometric constraints via a transformer to reconstruct 3D Gaussians, reducing the need for dense inputs. Human-3Diffusion (Xue et al., 2024) proposes a mutual refinement framework where 2D diffusion priors initialize 3D Gaussians, and 3D rendering feedback in turn refines the diffusion sampling, ensuring 3D consistency. Its successor, Gen-3Diffusion (Xue et al., 2025), generalizes this pipeline to generic object categories. AniGS (Qiu et al., 2025b) tackles the problem by first synthesizing multi-view canonical images and normal maps using a video generator, then treating reconstruction as a 4D problem solved via 4D Gaussian splatting.

Recently, methods explore specialized architectures for disentanglement or detailed reconstruction from a single image. Disco4D (Pang et al., 2025) proposes a clothing-body disentanglement framework that initializes separate Gaussians for each, uses diffusion to inpaint occluded regions, and guides optimization with clothing identity codes. SinGS (Wu et al., 2025) uses a kinematic diffusion model to generate plausible pose sequences from a single image and reconstructs an avatar via geometry-preserving splatting with semantic regularization. HumanRef-GS (Zhang et al., 2025) employs a reference-guided score distillation sampling framework, using pose and normal priors for initialization, enforcing multi-view consistency, and adopting isotropic Gaussians to reduce view-dependent artifacts, though it may still produce unnatural poses. LHM (Qiu et al., 2025a) introduces a generalizable model by fusing 3D geometric and image features with a Multimodal Body-Head Transformer (MBHT); although it achieves robust generalization and animation consistency rapidly, it still struggles with loose clothing. Subsequently, PERSONA (Sim and Moon, 2025) effectively handles loose garments by leveraging diffusion-generated videos and a hybrid SMPL-X/3DGS representation, modeling deformations via MLP-predicted offsets and employing balanced sampling and geometry-weighted optimization for identity-consistent, sharp renderings across different poses. However, PERSONA is incapable of simulating fabric physics; furthermore, the diffusion process is computationally expensive and requires a long time for preprocessing.

Monocular video-based human reconstruction has reduced the need for specialized equipment, but single-image reconstruction remains challenging due to incomplete data. While significant progress has been made in reconstruction quality and training efficiency through Gaussian representations and diffusion priors, challenges remain in handling extreme occlusions, achieving natural pose generation, and ensuring geometric consistency across novel poses.

### 3.2 Multi-view reconstruction

#### 3.2.1 Multi-view video processing

Multi-view video input offers richer spatial and temporal constraints, enabling high-fidelity reconstruction of dynamic human performances. A prominent line of work focuses on learning motion-dependent representations for robust animation. Animatable Gaussians (Li et al., 2024) learns a parametric template to guide splatting and uses a CNN to predict pose-dependent Gaussian maps, improving generalization. HuGS (Moreau et al., 2024) employs a coarse-to-fine deformation strategy, combining skinning with non-rigid refinements for real-time rendering. ASH (Pang et al., 2024) generates a motion-dependent mesh and texture via a deformation network, then predicts Gaussian parameters from the rendered texture. HiFi4G (Jiang et al., 2024b) proposes a dual-graph mechanism to balance motion priors and geometric updates, enabling high-fidelity performance capture. Its successor, DualGS (Jiang et al., 2024a), decouples motion and appearance into two Gaussian sets and uses a coarse-to-fine training strategy with advanced compression, achieving ultra-high compression rates suitable for VR.

To improve representation structure and training efficiency, another group of methods integrates explicit templates or geometric constraints. LayGA (Lin et al., 2024) uses a two-stage approach to model the body and clothing in separate layers, enabling virtual try-on. Animatable 3D Gaussian (Liu et al., 2024) demonstrates high reconstruction quality and efficiency for basketball players. MCGS (Zhang and Chen, 2024) replaces Marching Cubes with mesh-centric SDF enveloping and constrains Gaussians to mesh surfaces, ensuring accurate geometry-rendering correspondence. SK-GS (Wan et al., 2024) automatically discovers skeletal structures from dynamic scenes via superpoint clustering and part affinity. Hi-Fi Gaussian (Zhan et al., 2025) uses spatially-distributed MLPs on a template mesh to generate dynamic Gaussian parameters, enabling detailed pose-dependent deformation. TaoAvatar (Chen et al., 2025) builds a lightweight talking avatar by binding Gaussians to an extended SMPLX template, learning pose-dependent deformations with a StyleUNet distilled into an MLP, and adding learnable blend shapes for detail. These works showcase effective strategies for achieving high fidelity, efficient training, and realistic deformation from multi-view video.

#### 3.2.2 Reconstruction with multi-view images

Reconstruction from sparse multi-view images requires techniques that ensure view consistency and strong generalization despite limited input. Several approaches enhance cross-view consistency through geometry-aware mechanisms. GPS-Gaussian (Zheng et al., 2024) regresses 2D Gaussian parameter maps from input views and unprojects them into 3D, trained with depth supervision. GPS-Gaussian+ (Zhou et al., 2025) improves upon this by introducing an epipolar attention module for geometric consistency and removing the need for depth supervision via a rendering-based loss. Other methods integrate classical graphic representations for efficiency. UV Gaussians (Jiang et al., 2025) performs joint learning of mesh deformation and Gaussian texture in 2D UV space, leveraging 2D CNNs for feature extraction. GBC-Splat (Tu et al., 2025) reconstructs a fine-grained mesh by fusing occupancy and disparity, then anchors Gaussians to the mesh surface with adaptive subdivision for detail.

Another line of work targets high-fidelity performance capture under sparse views. CloCap-GS (Wang et al., 2025) aligns Gaussians with deforming body and clothing, jointly optimized under photometric constraints, and uses a physics-inspired cloth network to learn plausible dynamics. RoGSplat (Xiao et al., 2025) generates dense 3D prior points from SMPL vertices, fuses pixel and voxel features for coarse Gaussian prediction, and refines them with depth unprojection. These approaches highlight the integration of differentiable rendering with traditional graphic principles, enabling robust and generalizable multi-view human reconstruction even from limited image sets.

## 4 Conclusion and future directions

In this survey, we have provided a comprehensive overview of recent advances in reconstructing human avatars from both single-view and multi-view inputs. A prominent trend is the shift toward 3D Gaussian representations, which effectively balance high-fidelity rendering with computational efficiency. For monocular video, methods have evolved from learning canonical mappings with pose-refined deformations to incorporating hybrid Gaussians-on-mesh representations and occlusion-aware optimization, enabling fast training and real-time rendering. In the more constrained single-image setting, researchers have increasingly leveraged powerful diffusion priors and generative models to synthesize consistent geometry and appearance, though challenges in pose naturalness and occlusion handling remain. Multi-view approaches further exploit geometric constraints to achieve higher fidelity, through motion-dependent modeling, structured template-guided Gaussians, and improved cross-view consistency mechanisms. Collectively, these works demonstrate significant progress in creating photorealistic, animatable avatars while reducing reliance on expensive capture systems.

Despite these advances, several important challenges remain open for future research. (i) Unified and Editable Geometry Representation: Future work should develop hybrid representations that retain the rendering efficiency of 3D Gaussians while enabling direct extraction of editable, rigged meshes for broader animation and content creation applications. (ii) Robust Learning for Complex Clothing and Physics: Integrating physical simulation and cloth dynamics into reconstruction pipelines is essential to improve the realism and motion generalization of loose garments under monocular settings. (iii) Generalization and Few-Shot Learning: Advancing few-shot learning techniques (using stronger priors or diffusion models) will be critical for reducing input requirements and enhancing practicality for real-world applications.
